# Comparative and Phylogenetic Analysis of the Complete Chloroplast Genomes of Three *Paeonia* Section *Moutan* Species (Paeoniaceae)

**DOI:** 10.3389/fgene.2020.00980

**Published:** 2020-09-18

**Authors:** Liwei Wu, Liping Nie, Zhichao Xu, Pei Li, Yu Wang, Chunnian He, Jingyuan Song, Hui Yao

**Affiliations:** ^1^Key Laboratory of Bioactive Substances and Resources Utilization of Chinese Herbal Medicine, Ministry of Education, Institute of Medicinal Plant Development, Chinese Academy of Medical Sciences and Peking Union Medical College, Beijing, China; ^2^Engineering Research Center of Chinese Medicine Resources, Ministry of Education, Beijing, China

**Keywords:** section *Moutan*, chloroplast genome, comparative analysis, phylogeny, species relationship

## Abstract

Analysis of the relationships among wild species of section *Moutan* in the plant genus *Paeonia* has traditionally been problematic. Interspecies relationships cannot be effectively determined using phenotypic traits alone or through analysis of nuclear or chloroplast DNA fragments. Elucidation of complete chloroplast genome sequences will aid the identification and phylogeny of these species. In this study, the complete chloroplast genomes of three sect. *Moutan* plants were sequenced and analyzed. Comparative and phylogenetic analyses of the complete chloroplast genomes of all eight species of sect. *Moutan* were then conducted. The three complete chloroplast genomes gained in this study showed four-part annular structures, and the genome length, structure, GC content, codon usage, and gene distribution were highly similar. There was greater variation in the noncoding regions of the sequences than in the conserved protein-coding regions. Sequence variations in the small single copy (SSC) regions and large single copy (LSC) regions were considerably greater than those in the inverted repeat (IR) regions. Phylogenetic analysis revealed that the species of sect. *Moutan* clustered in one branch and then subdivided into smaller branches. As for the three complete chloroplast genome sequences obtained in this study, *Paeonia jishanensis* clustered with another *P. jishanensis* sequence from the GenBank database, *Paeonia qiui* clustered with *Paeonia rockii*, and *Paeonia delavayi* var. *lutea* clustered with *Paeonia ludlowii*. It was also found that the complete chloroplast genomes, LSC regions, and SSC regions all showed great abilities in identification and phylogenetic analysis of the species of sect. *Moutan*, while IRs regions and highly variable regions were not suitable for the species of sect. *Moutan*.

## Introduction

Section *Moutan* belongs to the genus *Paeonia*, which was previously classified in the family Ranunculaceae but is now under the family Paeoniaceae. In 1946, Stern divided the genus *Paeonia* into three groups, namely sect. *Moutan*, sect. *Paeonia* and sect. *Onaepia*. Section *Moutan* can be further divided into subsect. *Vaginatae* and subsect. *Delavayanae*, the latter consisting of the *Suffruticosa* and *Delavayi* groups (Stern, 1946). In the latest classification, sect. *Moutan* comprises eight species (Hong and Pan, 1999). Section *Moutan* plants are economically important ornamental plants known for their attractive flowers. Moreover, these plants also have high medicinal value as they exhibit anti-oxidant, anti-tumor, anti-pathogenic, anti-inflammatory, antidiabetic, analgesic, and anti-osteoporotic effects, and have been shown to exert protective effects against cardiovascular disease (Qi and Hu, 1993; Okubo et al., 2000; Kim et al., 2004; Abdel-Aty, 2007; Tsai et al., 2008; Zhang X. J. et al., 2008). These medicinal effects are attributed to the presence of monoterpene glucosides, flavonoids, tannins, triterpenoids, steroids, paeonols, phenolic acids, and other compounds in the plants (Okubo et al., 2000). In addition, the pollen of sect. *Moutan* plants contains various nutrients that can be used in the development of health and beauty products (Zhao, 2007).

Species of sect. *Moutan* are all subshrubs (Guo, 2002). All eight wild species of sec. *Moutan* are endemic to China (Hao et al., 2008): the wild species of sect. *Moutan* originated and evolved in China, which is also the birthplace of cultivated species of sect. *Moutan*. The wild species of sect. *Moutan* that are endemic to China are regarded as a valuable germplasm resource worldwide (Ji et al., 2012). Elucidating the relationships between species of sect. *Moutan* is crucial for understanding and harnessing the medicinal and ornamental properties of the different species. Progress has been made in studies on the relationships of wild species of sect. *Moutan*. However, the origin, genetic background, evolution, relationships, and classification systems of these species are different (Hong et al., 1998; Lin et al., 2004). According to classical taxonomy, using phenotypic traits alone to infer phylogenetic relationships between taxa with different genotypes is problematic. Moreover, different interpretations for the morphological variations in sect. *Moutan* species have been reported, and the different classification treatments applied vary largely (Zhang J. M. et al., 2008). Therefore, the relationships between wild species of sect. *Moutan* require further study and discussion. Molecular markers are a reliable alternative that are independent of morphological features, enabling the taxonomic challenges arising from the differences in interpretation of the morphological variations to be addressed. Molecular systematics have previously been used to study the evolutionary relationships among sect. *Moutan* species (Sang et al., 1997a,b; Zou et al., 1999; Zhao et al., 2004). However, the complex network evolution and polyploidy evolution of sect. *Moutan* species result that limited nuclear or chloroplast DNA fragments provide insufficient phylogenetic information to effectively solve interspecies relationships. Furthermore, the results of previous studies are inconsistent with each other (Sang et al., 1995; Sun and Hong, 2012).

The chloroplast genome is independent of the nuclear genome and corresponds to matrilineal inheritance with a separate transcription and transport system. Chloroplast genomes are very conservative (Jansen and Ruhlman, 2012) in terms of genome structure, gene sequence and gene type. Most chloroplast genomes of angiosperms have a circular tetrad structure existing as multiple copies of covalent, closed, circular double-stranded DNA. The circular tetrad structure comprises two inverted repeats (IRs), a large single copy (LSC), and a small single copy (SSC; Wang et al., 2012). Chloroplast genomes are typically 120–160 kb in length and are characterized by their small molecular weight, multiple copies, and slow molecular evolution (Shimada and Sugiura, 1991; Wakasugi et al., 2001; Kahlau et al., 2006). Since the chloroplast genomes of *Nicotiana tabacum* and *Marchantia polymorpha* were first reported in 1986 (Ohyama et al., 1986; Sugiura et al., 1986), the complete chloroplast genomes of various plants have been sequenced, and the structure, function, and expression of their genes have been studied. Chloroplast genome sequencing is increasingly used in the identification and investigation of molecular markers and phylogeny of medicinal plants (Wu et al., 2010; Kuang et al., 2011; Nock et al., 2011; Takano and Okada, 2011). Over the past years, chloroplast genomes have been shown to be an efficient tool in revealing phylogenetic relationships (Jansen et al., 2007), identifying close species as a super barcode (Chen et al., 2018; Park et al., 2018a), and developing chloroplast genetic engineering (Daniell et al., 2016).

In this study, the complete chloroplast genomes of three wild species of sect. *Moutan* were sequenced. A comparative analysis of the complete chloroplast genomes was then conducted. Phylogenetic analysis was performed by constructing phylogenetic trees based on different datasets of the chloroplast genomes of 16 species of the genus *Paeonia*, including all eight sect. *Moutan* species. Data obtained in this study provided a basis for the identification and investigation of the phylogenetic relationships of species of sect. *Moutan*.

## Materials and Methods

### DNA Sources

Fresh leaves of *Paeonia qiui*, *Paeonia jishanensis*, and *Paeonia delavayi* var. *lutea* were collected from Shennongjia in Hubei Province, Jiyuan in Henan Province and Shangri-la in Yunnan Province, respectively. The three species were identified by Professor Peigen Xiao and Professor Chunnian He from the Institute of Medicinal Plant Development (IMPLAD), Chinese Academy of Medical Sciences and Peking Union Medical College. Voucher specimens were deposited in the herbarium at IMPLAD.

### Total DNA Extraction and Sequencing

Total DNA was extracted using a DNeasy Plant Mini Kit (Qiagen, Germany). DNA concentration was determined using a microspectrophotometer (Nanodrop 2000, United States), and DNA quality was detected by 1% agarose gel electrophoresis. Illumina HiSeq X sequencing platform was used to construct a library with an insertion fragment of 500 bp. Paired-end sequencing was performed to obtain 150-bp sequences at both ends of each read.

### Assembly of Chloroplast Genome Sequences

Low-quality regions in the original sequencing data were removed using Trimmomatic software (Bolger et al., 2014). A local sequence comparison retrieval (BLASTn) database was constructed from the chloroplast genome sequences published in the National Center for Biological Information (NCBI). Clean reads were compared with this database, and mapped reads were extracted based on coverage and similarity. SOAPdenovo 2 (Luo et al., 2012) was used to assemble extracted reads into contigs. A scaffold of the chloroplast genome was constructed using SSPACE software (Boetzer et al., 2011). Gaps were filled using GapFiller (Nadalin et al., 2012).

### Chloroplast Genome Annotation and Structural Analysis

Dual Organellar GenoMe Annotator (Wyman et al., 2004) and chloroplast genome annotation, visualization, analysis, and genbank submission (CPGAVAS) (Liu et al., 2012) were used to initially annotate the sequences, and the annotations were then manually corrected. tRNAscan-SE software (Schattner et al., 2005) was used to annotate tRNA. Genes, introns and the boundaries of coding regions were compared with reference sequences. Chloroplast genome maps were generated using Organellar Genome DRAW v1.2 (Lohse et al., 2007) and then manually corrected. GC content was analyzed using MEGA 6.0 (Tamura et al., 2013). CodonW software (Sharp and Li, 1987) was adopted to analyze the relative synonymous codon usage (RSCU). The assembled complete chloroplast genome sequences of the three species of sect. *Moutan* were submitted to NCBI under the accession numbers MT210544 (*P. qiui*), MT210545 (*P. jishanensis*), and MT210546 (*P. delavayi* var. *lutea*).

### Structural Analysis of Repeats

REPuter software (Kurtz et al., 2001) was used to identify long repeat sequences of the chloroplast genomes. Microsatellite identification tool (MISA) software (Beier et al., 2017) was used to determine the type and number of simple sequence repeats (SSRs), employing the parameters used by Li et al. (2013). Completely repetitive SSRs were searched, and cyclically arranged or inversely complementary SSRs were treated as the same type.

### Analysis of Sequence Variations and Phylogenetic Relationships

Chloroplast genome sequences of all eight species of sect. *Moutan* were compared using the online genome comparison tool mVISTA (Frazer et al., 2004). Nucleic acid variation values of the three chloroplast genomes sequenced in this study were determined by DnaSP v5.10 (Librado and Rozas, 2009). The boundaries of four regions of chloroplast genomes were compared using IRscope (Ali et al., 2018). MAFFT version 5 software (Katoh and Standley, 2013) was used to compare the complete chloroplast genome sequences and LSC, SSC, and IRs regions of the chloroplast genomes of 16 species of the genus *Paeonia* (Supplementary Table S1). IQTREE software (Nguyen et al., 2015) and MrModeltest 2.3 (Nylander, 2004) were utilized to select tree models. Maximum likelihood (ML) and Bayesian inference (BI) phylogenetic trees were constructed based on the complete chloroplast genomes and LSC, SSC, and IRs regions using the program IQTREE and MrBayes v3.2.7 (Ronquist and Huelsenbeck, 2003), respectively. MEGA 6.0 (Tamura et al., 2013) was used to construct ML phylogenetic trees based on 19 highly variable regions, which were selected by the percent identity of the complete chloroplast genomes using mVISTA (Frazer et al., 2004). The positions of these highly variable regions were shown in Supplementary Table S2.

## Results

### Molecular Features of the Chloroplast Genomes

**FIGURE 1:**
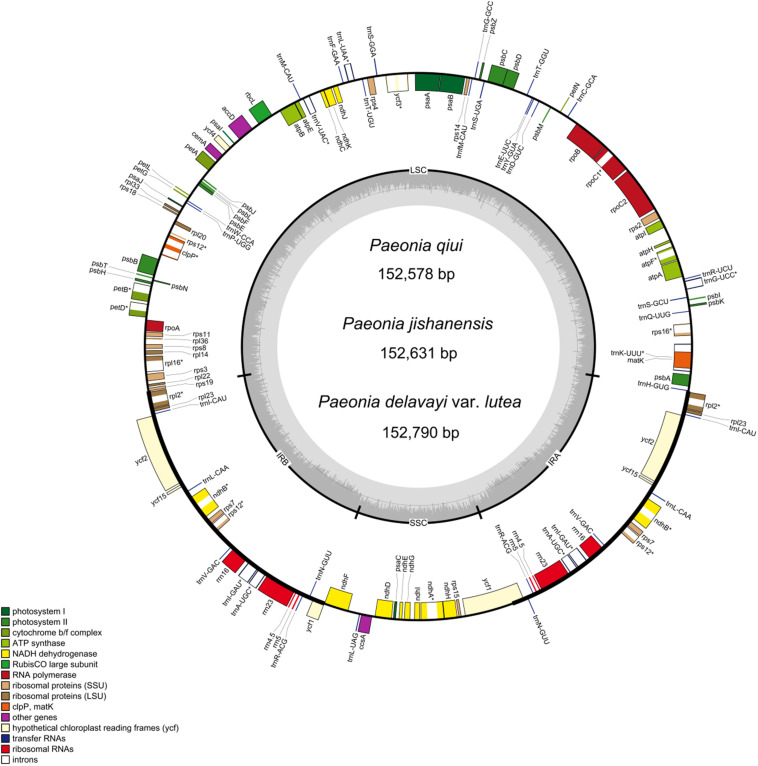
Gene maps of the complete chloroplast genomes of the three sect. *Moutan* species. Genes on the inside of the circle are transcribed clockwise, while those outside are transcribed counter clockwise. The darker gray in the inner circle corresponds to GC content, whereas the lighter gray corresponds to AT content. *matK* in *P. delavayi* var. *lutea* is a pseudo gene.

The complete chloroplast genomes of three species of sect. *Moutan* show a common tetrad structure comprising two IRs (25,645–25,648 bp), an LSC (84,242–84,462 bp), and an SSC (17,032–17,046 bp; Figure 1). The total lengths of the chloroplast genomes were 152,578 bp (*P. qiui*), 152,631 bp (*P. jishanensis*) and 152,790 bp (*P. delavayi* var. *lutea*), while the total GC content ranged from 38.35 to 38.42%. Moreover, GC content in different regions was unbalanced; the IR regions had the highest GC content (43.06–43.10%) among the four sections, followed by the LSC (36.64–36.73%) and SSC regions (32.59–32.73%) (Table 1).

**TABLE 1:** Statistics for assembly of the three chloroplast genomes.

**Latin name**	**Gene size (bp)**	**IRs**		**LSC**		**SSC**		**GC Content (%)**
		**size (bp)**	**GC Content (%)**	**size (bp)**	**GC Content (%)**	**size (bp)**	**GC Content (%)**	
*P. qiui*	152,578	25,646	43.06	84,242	36.66	17,044	32.62	38.36
*P. jishanensis*	152,631	25,645	43.06	84,295	36.64	17,046	32.59	38.35
*P. delavayi* var. *lutea*	152,790	25,648	43.10	84,462	36.73	17,032	32.73	38.42

The chloroplast genome structure and gene composition of sect. *Moutan* species could be divided into four categories: genes related to photosynthesis, genes related to self-replication, protein-coding genes with unknown functions, and other genes (e.g., mature enzyme gene *matK* and cystic protein gene *cemA*). A total of 84 protein-coding genes were annotated in *P. qiui* and *P. jishanensis*, while 83 protein-coding genes were annotated in *P. delavayi* var. *lutea*. Compared with other species, the polyT repeat region of *matK* gene in *P. delavayi* var. *lutea* lost one T base, and the frame shift caused stop codon to appear prematurely, so the gene was annotated as a pseudo gene. Thirty-seven tRNA genes and 8 rRNA genes were annotated in the three species. Seven protein-coding genes (*rpl2*, *rpl23*, *ycf2*, *ycf15*, *ndhB*, *rps7*, and *rps12*), seven tRNAs (*trnI-CAU*, *trnL-CAA*, *trnV-GAC*, *trnI-GAU*, *trnA-UGC*, *trnR-ACG*, and *trnN-GUU*) and four rRNAs (*rrn16*, *rrn23*, *rrn4.5*, and *rrn5*) were located in the IR regions. Among the protein-coding genes, 18 genes contained introns; three of these genes (*clpP, rps12*, and *ycf3*) had two introns, and 15 genes had only one intron. The gene *rps12*, which contained two introns, is a trans-splicing gene with the 5’ exon in the LSC region and the 3’ exon in the IR region (Table 2).

**TABLE 2:** Genes with introns in the chloroplast genomes of *P. qiui*, *P. jishanensis* and *P. delavayi* var. *lutea* as well as the lengths of the exons and introns.

**Gene**	**Location**	***P. qiui***					***P. jishanensis***					***P. delavayi* var. *lutea***				
		**Exon I (bp)**	**Intron I (bp)**	**Exon II (bp)**	**Intron II (bp)**	**Exon III (bp)**	**Exon I (bp)**	**Intron I (bp)**	**Exon II (bp)**	**Intron II (bp)**	**Exon III (bp)**	**Exon I (bp)**	**Intron I (bp)**	**Exon II (bp)**	**Intron II (bp)**	**Exon III (bp)**
*atpF*	LSC	159	696	411			159	696	411			159	696	411		
*clpP*	LSC	69	672	291	657	228	69	671	291	657	228	69	676	291	658	228
*ndhA*	SSC	543	1010	540			543	1011	540			543	1013	540		
*ndhB*	IR	777	682	756			777	682	756			777	682	756		
*petB*	LSC	6	760	651			6	758	651			6	768	651		
*petD*	LSC	9	694	474			9	694	474			9	694	474		
*rpl16*	LSC	9	1012	399			9	1018	399			9	1011	399		
*rpl2*	IR	393	668	435			393	668	435			393	668	435		
*rpoC1*	LSC	436	703	1616			436	701	1616			436	694	1616		
*rps12*	LSC/IR	114	–	232	535	26	114	–	232	535	26	114	–	232	535	26
*rps16*	LSC	39	818	234			39	818	234			39	819	234		
*trnA-UGC*	IR	38	717	35			38	717	35			38	717	35		
*trnG-UCC*	LSC	34	696	48			34	696	48			34	691	48		
*trnI-GAU*	IR	42	933	35			42	933	35			42	933	35		
*trnK-UUU*	LSC	37	2442	35			37	2447	35			37	2459	35		
*trnL-UAA*	LSC	37	521	50			37	522	50			37	510	50		
*trnV-UAC*	LSC	39	573	37			39	573	37			39	576	37		
*ycf3*	LSC	126	718	228	763	153	126	720	228	763	153	126	721	228	763	153

### Relative Synonymous Codon Usage Analysis of the Chloroplast Genomes

**FIGURE 2:**
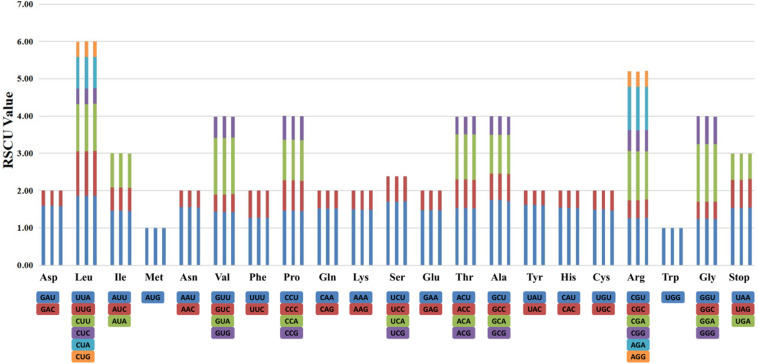
Codon distribution of 20 amino acid and stop codons in all protein-coding genes of the chloroplast genomes of three sect. *Moutan* species. The order of every three columns is *P. qiui*, *P. jishanensis*, and *P. delavayi* var. *lutea*, respectively.

The RSCU of the chloroplast genomes of species of sect. *Moutan* was calculated using all protein-coding genes. The RSCU value is the ratio of the frequency of use of a particular codon to the expected frequency. It enables the detection of synonymous codons that do not uniformly occur in the coding sequence. Codons with no preference value are set to 1.00. The actual usage of codons with an RSCU value >1.00 is higher than expected, and that of codons with an RSCU value <1.00 is lower than expected. Among all amino acids, leucine (Leu) had the highest number of codons in all protein-coding gene sequences in the chloroplast genomes. The amino acids with a total codon number <1000 were methionine (Met), tyrosine (Tyr), histidine (His), glutamine (Gln), cysteine (Cys), tryptophan (Trp), serine (Ser), and terminator (TER); Cys had the lowest number codons (Supplementary Table S3). Figure 2 shows the codon contents of 20 amino acids and stop codons of all protein-coding genes in the chloroplast genomes of the three species of sect. *Moutan* sequenced in this study.

### Long Repeat Sequence and SSR Analyses

**FIGURE 3:**
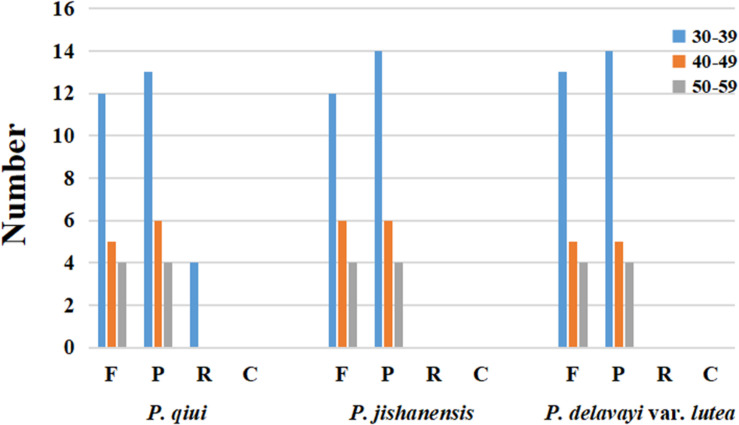
Repeat sequences in three chloroplast genomes. REPuter was used to identify repeat sequences with length ≥30 bp and sequences identified ≥90% in the chloroplast genomes. F, P, R, and C indicate the repeat types F (forward), P (palindrome), R (reverse), and C (complement). Repeats with different lengths are indicated in different colors.

Long repeat sequences are classified as forward (F), palindrome (P), reverse (R), or complement (C). For all repeat types, the repeat length is ≥30 bp and sequence similarity is ≥90%. In the chloroplast genome of *P. qiui*, there were 21 F repeats, 23 P repeats, and 4 R repeats. Furthermore, 22 F repeats and 24 P repeats were present in the chloroplast genome of *P. jishanensis*, while the chloroplast genome of *P. delavayi* var. *lutea* contained 22 F repeats and 23 P repeats. No C repeats were found in the chloroplast genomes of the three sect. *Moutan* species, and no R repeats were identified in the chloroplast genomes of *P. jishanensis* and *P. delavayi* var. *lutea*. The length of the repeat sequences that were found predominantly ranged from 30 to 39 bp. R repeat sequences of the *P. qiui* chloroplast genome only contained 30–39 bp (Figure 3).

Simple sequence repeats in the chloroplast genomes have abundant polymorphisms, and they are an efficient molecular marker (Tang et al., 2010). In this study, 73, 72, and 61 SSRs were identified in the chloroplast genomes of *P. qiui*, *P. jishanensis*, and *P. delavayi* var. *lutea*, respectively. In addition, the base composition of the repeating motifs from mononucleotide repeats to trinucleotide repeats had a certain base preference, mainly repeating motifs rich in A–T. In these SSRs, mononucleotide repeats were the most abundant, being found 49, 47, and 39 times in the chloroplast genomes of *P. qiui*, *P. jishanensis*, and *P. delavayi* var. *lutea*, respectively. A/T repeats were the most common mononucleotide repeats (93.9, 97.9, and 100% for *P. qiui*, *P. jishanensis*, and *P. delavayi* var. *lutea*, respectively). Dinucleotide repeat sequences predominantly comprised AT/AT repeats (91.7, 92.3, and 92.3% for *P. qiui*, *P. jishanensis*, and *P. delavayi* var. *lutea*, respectively), and all trinucleotide repeats were AAT/ATT. This was consistent with A–T enrichment in the complete chloroplast genomes of sect. *Moutan* species (61.6, 61.7, and 61.6% for *P. qiui*, *P. jishanensis*, and *P. delavayi* var. *lutea*, respectively) (Table 3).

**TABLE 3:** Types and amounts of SSRs in the three chloroplast genomes.

**SSR Type**	**Repeat Unit**	**Amount**			**Ratio (%)**		
		***P. qiui***	***P. jishanensis***	***P. delavayi* var. *lutea***	***P. qiui***	***P. jishanensis***	***P. delavayi* var. *lutea***
Mono	A/T	46	46	39	93.9	97.9	100
	C/G	3	1	0	6.1	2.1	0
Di	AG/CT	1	1	1	8.3	7.7	7.7
	AT/AT	11	12	12	91.7	92.3	92.3
Tri	AAT/ATT	7	6	5	100	100	100
Tetra	AAAC/GTTT	1	1	1	20	20	25
	AAAG/CTTT	1	1	0	20	20	0
	AAAT/ATTT	2	2	2	40	40	50
	AGAT/ATCT	1	1	1	20	20	25
Penta	AATAT/ATATT	0	1	0	0	100	0

### Comparative Analysis of the Chloroplast Genomes

**FIGURE 4:**
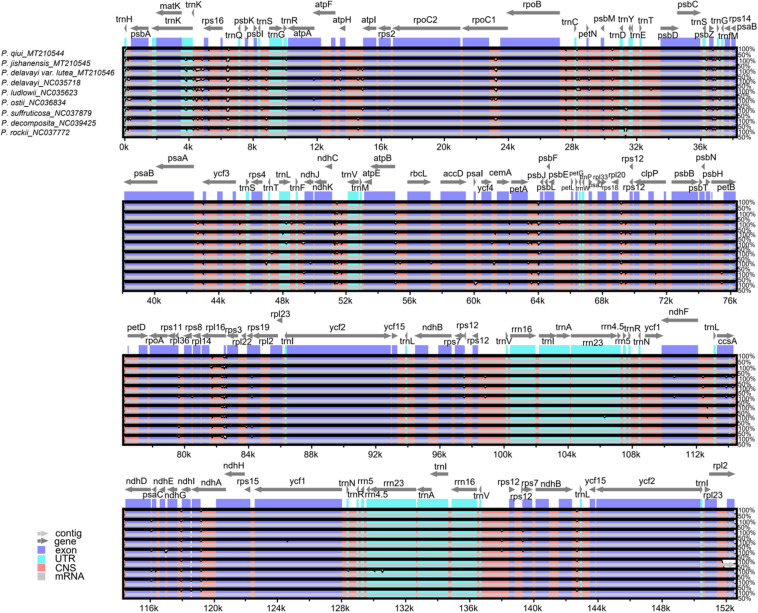
Global alignment of chloroplast genomes of all sect. *Moutan* species. Gray arrows and thick black lines above the alignment indicate genes with their orientation and the position of the IRs, respectively. A cutoff of 70% identity was used for the plots, and the Y-scale represents the percent identity ranging from 50 to 100%.

In this study, the complete chloroplast genomes of all species of sect. *Moutan* were compared using mVISTA (Frazer et al., 2004) with the *P. qiui* genome as the reference genome (Figure 4). There were more variations in the noncoding regions of the sequences than in the conserved protein-coding regions. Variations in the SSC and LSC regions were considerably greater than those in the IR regions, while the rRNA genes were highly conserved with almost no variation. As shown in Figure 4, the genes with large variations included *trnK*, *trnR*, *psbZ*, *ycf3*, *rps3*, and *rps19*, whereas the other genes had a very high degree of conservation (most had >90% similarity). Variations in intergenic regions were notably greater than those in gene regions; such intergenic regions included *trnK-rps16*, *rps16-trnQ*, *rpoC1-rpoB*, *rpoB-trnC*, *psbM-trnD*, *psbZ-trnG*, *ndhC-trnV*, *atpB-rbcL*, *petA-psbJ*, *rpl20-rps12*, *rpl16-rps3*, *ndhG-ndhI*, *ndhA-ndhH*, and *ndhB-trnL*.

**FIGURE 5:**
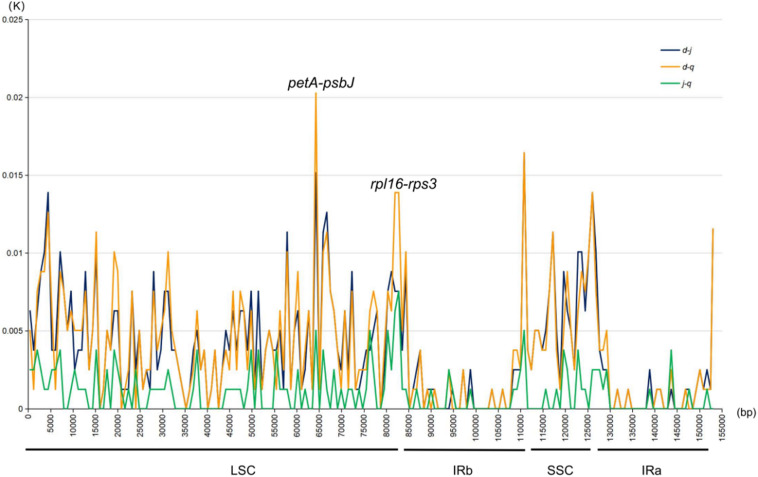
Nucleic acid variation information of chloroplast genomes of sect. *Moutan* species. d: *P. delavayi* var. *lutea*; j: *P. jishanensis*; q: *P. qiui*.

DnaSP (Librado and Rozas, 2009) was used to analyze and detect highly variable regions in the chloroplast genomes sequenced in this study. The K value was calculated by pairwise comparisons to determine variations at the sequence level (Figure 5). Variation in the IR regions of the chloroplast genomes was markedly lower than that in the LSC and SSC regions, consistent with the mVISTA results. Furthermore, the K value was generally below 0.005. In the LSC regions, two pairwise-comparison peaks with a K value >0.005 were particularly prominent. As shown in the mVISTA map and specific sites, the two peaks were *petA-psbJ* and *rpl16-rps3*. The average K value was 0.00372 between *P. delavayi* var. *lutea* and *P. jishanensis*, 0.00378 between *P. delavayi* var. *lutea* and *P. qiui*, and 0.00100 between *P. jishanensis* and *P. qiui*. Thus, the largest nucleic acid variation was observed between *P. delavayi* var. *lutea* and *P. qiui*, followed by that between *P. delavayi* var. *lutea* and *P. jishanensis*, and that between *P. jishanensis* and *P. qiui*.

**FIGURE 6:**
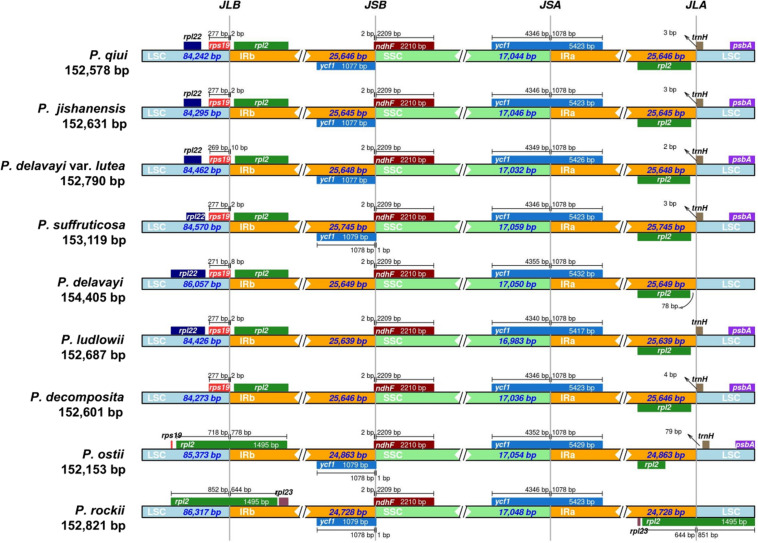
Comparison of the borders of LSC, SSC, and IR regions among all sect. *Moutan* species chloroplast genomes. Number above the gene features means the distance between the ends of genes and the border sites. These features are not to scale. JLB, junction of LSC/IRb; JSB, junction of IRb/SSC; JSA, junction of SSC/IRa; JLA, junction of IRa/LSC.

The boundaries of the four regions of chloroplast genomes of all species of sect. *Moutan* were comprehensively compared. At the junctions, the gene positions in the boundary regions of the three chloroplast genomes sequenced in this study and that of *P. suffruticosa* were very similar, whereas the chloroplast genomes of the five other species of sect. *Moutan* were different (Figure 6). In addition, it could be seen from the figure that the genome length of *P. delavayi* was much longer than that of its variety *P. delavayi* var. *lutea*, mainly because the length of the LSC region of *P. delavayi* was about 1600 bp longer than that of *P. delavayi* var. *lutea*. There were one more *infA*, *trnP-GGG*, *trnT-GGU* and *trnM-CAU* genes in the LSC region of *P. delavayi* than those in *P. delavayi* var. *lutea*.

### Phylogenetic Analysis

**FIGURE 7:**
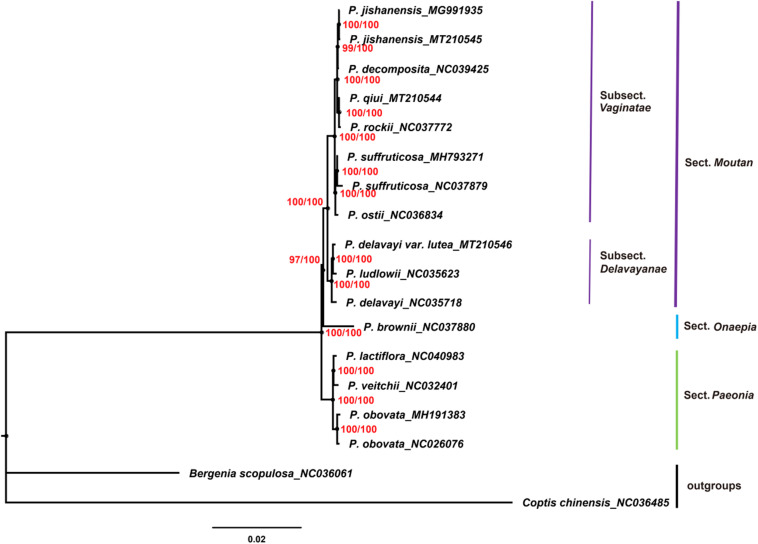
Phylogenetic trees constructed using Maximum Likelihood (ML) and Bayesian Inference (BI) methods based on the complete chloroplast genome sequences of 16 *Paeonia* species, including all eight species of sect. *Moutan*. Red numbers at nodes are values for bootstrap support (ML/BI).

**FIGURE 8:**
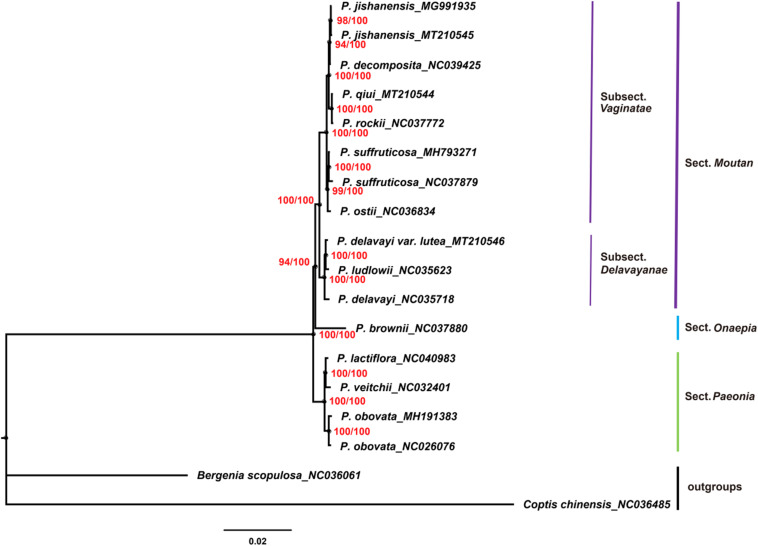
Phylogenetic trees constructed using ML and BI methods based on the LSC regions of the chloroplast genomes of 16 *Paeonia* species, including all eight species of sect. *Moutan*. Red numbers at nodes are values for bootstrap support (ML/BI).

**FIGURE 9:**
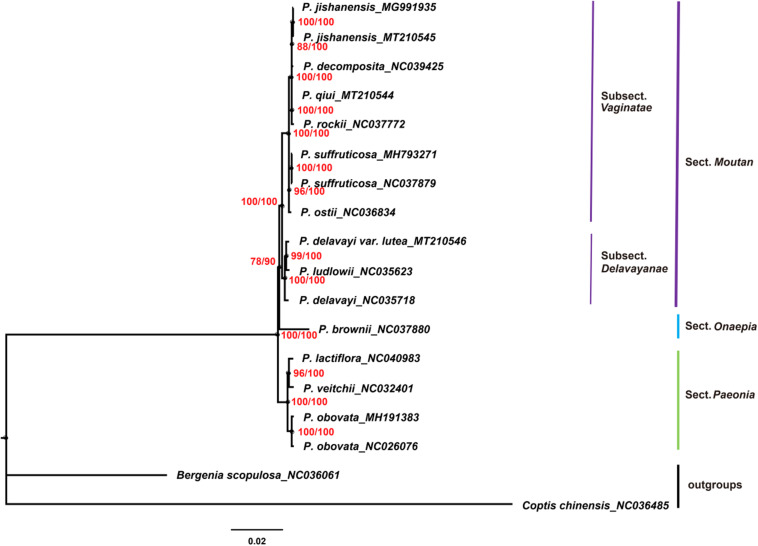
Phylogenetic trees constructed using ML and BI methods based on the SSC regions of the chloroplast genomes of 16 *Paeonia* species, including all eight species of sect. *Moutan*. Red numbers at nodes are values for bootstrap support (ML/BI).

Chloroplast genomes play an important role in phylogenetic studies (Zhang et al., 2011; Hu et al., 2016). In the current study, the complete chloroplast genome sequences and LSC, SSC, and IRs regions of the chloroplast genomes of 16 species of the genus *Paeonia*, including all eight wild species of sect. *Moutan*, were used to construct ML and BI trees, with *Bergenia scopulosa* and *Coptis chinensis* as the outgroups. The two phylogenetic analyses (ML and BI) revealed congruent topologies based on the complete chloroplast genomes, LSC regions and SSC regions, and all of the nodes in the phylogenetic trees had high bootstrap support values (Figures 7–9). The resulting phylogenetic trees demonstrated that species of sect. *Moutan* were located on one branch, whereas species of the sect. *Onaepia* and sect. *Paeonia* were located on another branch. Species of subsect. *Vaginatae* and subsect. *Delavayanae* in sect. *Moutan* clustered in different branches, and the bootstrap support values for these were 100%. For the three chloroplast genomes sequenced in this study, *P. jishanensis* (GenBank accession no. MT210545) clustered with another *P. jishanensis* sequence obtained from the GenBank database, *P. qiui* (MT210544) clustered with *P. rockii*, and *P. delavayi* var. *lutea* (MT210546) clustered with *P. ludlowii*. Bootstrap support rates were all >95%. However, the two phylogenetic analyses (ML and BI) revealed incongruent topologies based on the IRs regions (Supplementary Figure S1). In the phylogenetic trees based on IRs regions, the two sequences of *P. suffruticosa* did not cluster together, and in subsect. *Delavayanae*, *P. delavayi* clustered with *P. ludlowii* and then with *P. delavayi* var. *lutea*, which was different from that based on complete chloroplast genomes. In addition, some of the nodes had very low bootstrap support values. It showed that IRs regions were not suitable for the identification and phylogenetic analysis of the species of sect. *Moutan*.

Then 19 highly variable regions of chloroplast genomes were used to construct ML trees (Supplementary Figure S2). Most of the bootstrap support values of the nodes in the phylogenetic trees were much lower than that based on complete chloroplast genomes. Species of sect. *Moutan* did not cluster in one branch in some phylogenetic trees, such as the trees based on *psbZ*, *rps19*, *trnR*, *ndhG-ndhI*, *psbM-trnD*, *rpl16-rps3*, *rpoC1-rpoB*, and *rps16-trnQ*. In the phylogenetic trees based on *ycf3*, *ndhB-trnL*, *psbZ-trnG*, *rpl20-rps12*, and *trnK-rps16*, species of subsect. *Vaginatae* and subsect. *Delavayanae* did not cluster in one branch, respectively. The other highly variable regions were also not suitable for the identification and phylogenetic analysis of the species of *sect. Moutan*.

## Discussion

### Analysis of the Chloroplast Genomes of Sect. *Moutan* Species

In the current study, the GC content distribution in the chloroplast genomes of the three species of sect. *Moutan* was the same as that reported for most other angiosperms (Xiang et al., 2016; Zhou et al., 2017); the IR regions had the highest GC content among the four regions, followed by the LSC and SSC regions. The high GC content in the IR regions may be attributed to the fact that these regions contain rRNAs with low A/T content in the chloroplast genome, including *rrna4.5*, *rrna5*, *rrna23*, and *rrna16*. In all types of SSR in this study, A and T were the most often-used bases. Chloroplast genome SSRs are typically composed of polyA or polyT repeats, as a result of the A/T base preference of chloroplast genomes (Qian et al., 2013). These SSRs not only have the advantages of an abundant number of markers, codominant inheritance and high repeatability, but also have the characteristics of a simple structure, the single-parent inheritance of chloroplast genomes and are relatively conservative (Yang et al., 2014). Chloroplast genome SSRs have been widely used in species identification, phylogenetic analysis, population genetic structure and system geography of several species (Park et al., 2018b).

In the current study, a common feature of the chloroplast genomes of sect. *Moutan* species was that the IR regions were substantially more conserved than the LSC and SSC regions. *rrn4.5*, *rrn5*, *rrn16*, and *rrn23* were the most conserved sequence regions in the IR regions. In addition, the degree of variation in noncoding regions was considerably greater than that in coding regions. The evolution rate of coding regions is slow and thus these regions are suitable for phylogenetic analysis at high levels of taxonomic hierarchy, such as families and orders (Li et al., 2012). In contrast, the sequence of noncoding regions rapidly evolves, cannot encode proteins, and contains an abundance of variation information. Noncoding regions are therefore suitable for phylogenetic analysis at low levels of taxonomic hierarchy, such as genera (Shaw et al., 2007). Noncoding regions can be subdivided into introns and intergenic regions and can be used for molecular identification of subspecies (Shaw et al., 2007). In the current study, 18 genes contained introns; three of these genes (*clpP*, *rps12*, and *ycf3*) contained two introns, and 15 genes contained only one intron. Furthermore, the intergenic regions of the sect. *Moutan* chloroplast genomes were variable.

The study of chloroplast genomes is highly relevant for revealing the structure and origin of chloroplast DNA, plant molecular markers and species relationships (Tang et al., 2011). Chloroplast genome sequencing and phylogenetic analysis of species of sect. *Moutan* can enrich the number of chloroplast genome sequences and lay the foundation for species identification, phylogenetic relationship, breeding of improved varieties and sustainable exploitation of plant resources. Furthermore, this sequencing and analysis also provides a theoretical basis for studying chloroplast genetic engineering in species of sect. *Moutan*.

### Phylogenetic Analysis of Sect. *Moutan* Species and Chloroplast Genome Super Barcode

Species of sect. *Moutan* are economically important ornamental plants that are also commonly used as medicinal plants. However, the phylogenetic relationships and taxonomic systems of wild species of sect. *Moutan* were different in previous studies (Lin et al., 2004), and this could affect their applications. Plant classification and identification have been based on morphological evidence for a long time. However, morphological traits are easily affected by the environment, and convergence and parallel evolution often occur (Xing et al., 2013). DNA studies can provide reliable molecular evidence for phylogenetic evolution of species and identification of similar species within a genus. Numerous genes, such as the nuclear genes ITS, *Adh* and GPAT and the chloroplast genes *matK*, *psbA-trnH*, and *trnL(UAA)-trnf(GAA)*, have been applied to phylogenetic relationship studies of species of sect. *Moutan* (Sang et al., 1995, 1997a,b; Ferguson and Sang, 2001; Tank and Sang, 2001; Sang, 2002; Sun and Hong, 2012). Although these studies have advanced our understanding of the relationships among species of sect. *Moutan*, the results of different studies are not completely consistent.

In the current study, phylogenetic trees were constructed based on the complete chloroplast genomes and LSC, SSC, and IRs regions of the chloroplast genomes of 16 species of the genus *Paeonia*, including all eight wild species of sect. *Moutan*. Among them, complete chloroplast genomes, LSC regions and SSC regions showed good abilities in the phylogenetic analysis of Sect. *Moutan* species. Species of sect. *Moutan*, sect. *Onaepia* and sect. *Paeonia* clustered in large distinct branches of the phylogenetic tree, and the two subgroups of sect. *Moutan* were further subdivided into different branches. In subsect. *Vaginatae*, *P. jishanensis*, *P. decomposita*, *P. qiui*, and *P. rockii* clustered in one small branch, while *P. ostii* and *P. suffruticosa* clustered in a different small branch, consistent with previous studies (Zhao et al., 2004; Zhao, 2007). Previous studies (Zou et al., 1999; Tank and Sang, 2001; Feng et al., 2015) showed that *P. decomposita* was related to *P. rockii*, and *P. qiui* was related to *P. jishanensis*; however, the current study indicated that *P. decomposita* was related to *P. jishanensis*, while *P. rockii* was related to *P. qiui*. Wang (1996) and Zhang J. M. et al. (2008) used gene fragments of the chloroplast genomes of sect. *Moutan* species to analyze their relationships and found that *P. decomposita* and *P. jishanensis* were closely related and clustered together in the phylogenetic tree. This is consistent with the results of the present study. Furthermore, a pathway proposed by Zhou and Yao (2002) for the phylogenetic evolution of subsect. *Vaginatae* based on morphological traits produced the same conclusion as the current study. In subsect. *Delavayanae*, *P. delavayi* var. *lutea* clustered with *P. ludlowii* and then with *P. delavayi*. This demonstrated that *P. delavayi* var. *lutea* and *P. ludlowii* are more closely related to each other than to *P. delavayi*. This is consistent with the finding that *P. delavayi* var. *lutea* used to be an independent species named *P. lutea* and that *P. ludlowii* was a variety of this species known as *P. lutea* var. *ludlowii* (Stern and Taylor, 1951). Feng et al. (2015) came to the same conclusion using chloroplast genes *psbA-trnH*. However, IRs regions were not suitable for the identification and phylogenetic analysis of the species of sect. *Moutan*. It was mainly because IRs regions were more conserved and had less variations, just as described above.

In addition, highly variable regions of chloroplast genomes were used to analyze their phylogenetic relationships, but most of the support values in the phylogenetic trees were very low, and none of them effectively resolved relationships among the sect. *Moutan* species. The relationships among sect. *Moutan* species were resolved with very high support values in the phylogenetic trees based on complete chloroplast genomes. The complete chloroplast genomes were better than the highly variable regions in the analysis of phylogenetic relationships of the sect. *Moutan* species. It was mainly due to the inadequate variations provided by a limited number of DNA loci (Xu et al., 2015), while the complete chloroplast genome could provide sufficient informative sites, which can help to clarify the relationships of intractable groups at low taxonomic levels (Yang et al., 2013; Li et al., 2017; Yu et al., 2017). In fact, chloroplast genomes have been proposed as super barcodes for species identification (Li et al., 2015). Super barcodes overcome many limitations of traditional barcodes (Parks et al., 2009; Steele and Pires, 2011; Coissac et al., 2016). Zhu et al. (2018) experimentally demonstrated that complete chloroplast genome sequences have higher resolution than DNA barcodes or highly variable regions of chloroplast genomes and can be used to identify related species, consistent with the current study. Super barcodes have been successfully used to identify species and individuals (Doorduin et al., 2011; Kane et al., 2012; Chen et al., 2018; Ma et al., 2018). The phylogenetic trees constructed in this study demonstrated that complete chloroplast genome sequences can also be used as a reference for the identification of species of sect. *Moutan*. With the rapid development of sequencing technology and analytical methods, chloroplast genome assembly sequencing is predicted to become widely used as a super barcode.

## Conclusion

The chloroplast genome structure and gene content of species of section *Moutan* in the genus *Paeonia* were relatively conserved, while the GC content and variations in LSC, SSC, and IRs regions of the sequences were different. In addition, abundant repetitive sequences were identified, and their nucleotide composition was analyzed. The phylogenetic analysis illustrated that the two subgroups of sect. *Moutan* clustered in different branches. In subsect. *Vaginatae*, *P. jishanensis*, *P. decomposita*, *P. qiui*, and *P. rockii* clustered in a small branch, while *P. ostii* and *P. suffruticosa* clustered in a different branch. *P. decomposita* was found to be related to *P. jishanensis*, and *P. rockii* was related to *P. qiui*. In subsect. *Delavayanae*, *P. delavayi* var. *lutea* and *P. ludlowii* were more closely related to each other than to *P. delavayi*. Furthermore, it was found that the complete chloroplast genomes, LSC regions and SSC regions had higher discrimination than IRs regions and highly variable regions for the species of sect. *Moutan*, and the complete chloroplast genomes could also be used as a super barcode for the identification of species of sect. *Moutan*.

## Data Availability Statement

The assembled chloroplast genomes of *P. qiui*, *P. jishanensis*, and *P. delavayi* var. *lutea* were deposited in GenBank with the accession numbers MT210544, MT210545, and MT210546. The sequences are available on NCBI now https://www.ncbi.nlm.nih.gov/nuccore/MT210544.1/, https://www.ncbi.nlm.nih.gov/nuccore/MT210545, and https://www.ncbi.nlm.nih.gov/nuccore/MT210546.

## Author Contributions

LW, LN, and PL performed the experiments. ZX and JS assembled the sequences. LW and LN analyzed the data. LW wrote the manuscript. LN, YW, and CH collected the plant material. HY conceived the research and revised the manuscript. All authors read and approved the final manuscript.

## Conflict of Interest

The authors declare that the research was conducted in the absence of any commercial or financial relationships that could be construed as a potential conflict of interest.

## Supplementary Material

FIGURE S1 Phylogenetic trees constructed using Maximum Likelihood (ML) and Bayesian Inference (BI) methods based on the IRs regions of the chloroplast genomes of 16 *Paeonia* species.

FIGURE S2 Phylogenetic trees constructed using the ML method based on 19 highly variable regions of chloroplast genomes of 16 *Paeonia* species.

TABLE S1 Information on the 16 *Paeonia* species.

TABLE S2 The positions of the 19 highly variable regions.

TABLE S3-1 Codons in chloroplast genome of *P. qiui*.

TABLE S3-2 Codons in chloroplast genome of *P. jishanensis*.

TABLE S3-3 Codons in chloroplast genome of *P. delavayi* var. *lutea*.
