# Supplementary material for: Comparative and Phylogenetic Analysis of the Complete Chloroplast Genomes of Three Paeonia Section Moutan Species (Paeoniaceae)
Source: Front Genet. 2020 Sep 18;11:980. doi: 10.3389/fgene.2020.00980 (PMC7533573; doi:10.3389/fgene.2020.00980)
Supplement: FIGURE S1 — Phylogenetic trees constructed using Maximum Likelihood (ML) and Bayesian Inference (BI) methods based on the IRs regions of the chloroplast genomes of 16 Paeonia species. [file Data_Sheet_1.pdf]

Supplementary Material

1 Supplementary Figures and Tables

1.1 Supplementary Figures

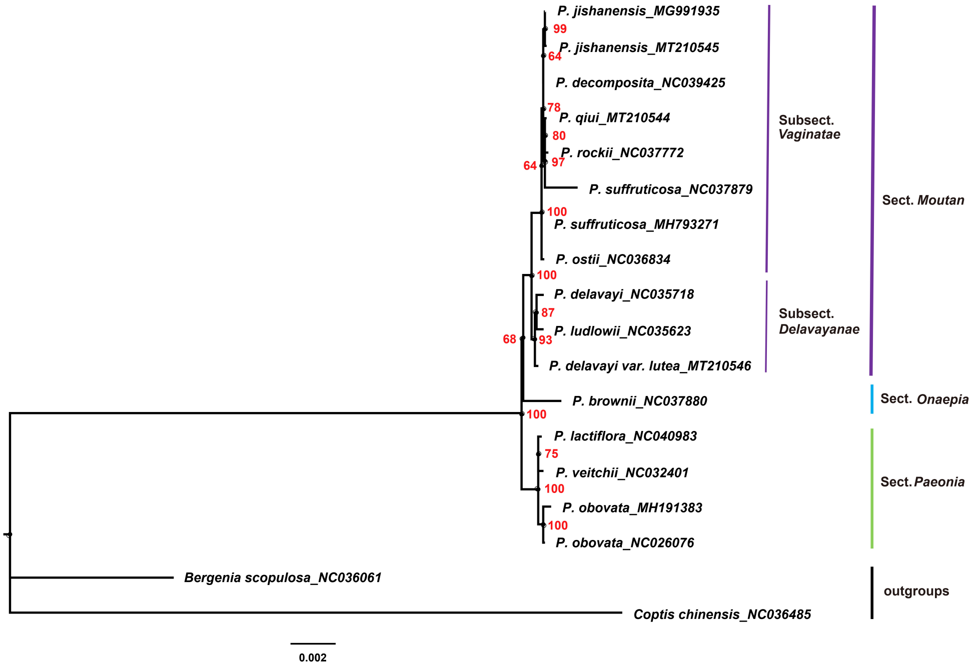

Supplementary Figure 1-1. Phylogenetic trees constructed using Maximum Likelihood (ML) method based on the IRs regions of the chloroplast genomes of 16 *Paeonia* species, including all eight species of sect. *Moutan*. Red numbers at nodes are values for bootstrap support.

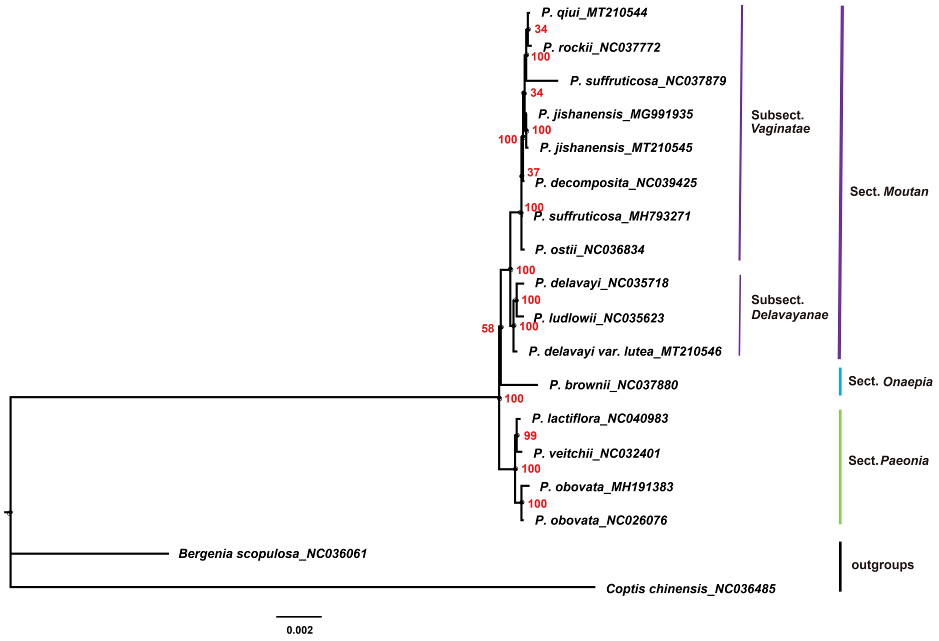

Supplementary Figure 1-2. Phylogenetic trees constructed using Bayesian Inference (BI) method based on the IRs regions of the chloroplast genomes of 16 *Paeonia* species, including all eight species of sect. *Moutan*. Red numbers at nodes are values for bootstrap support.

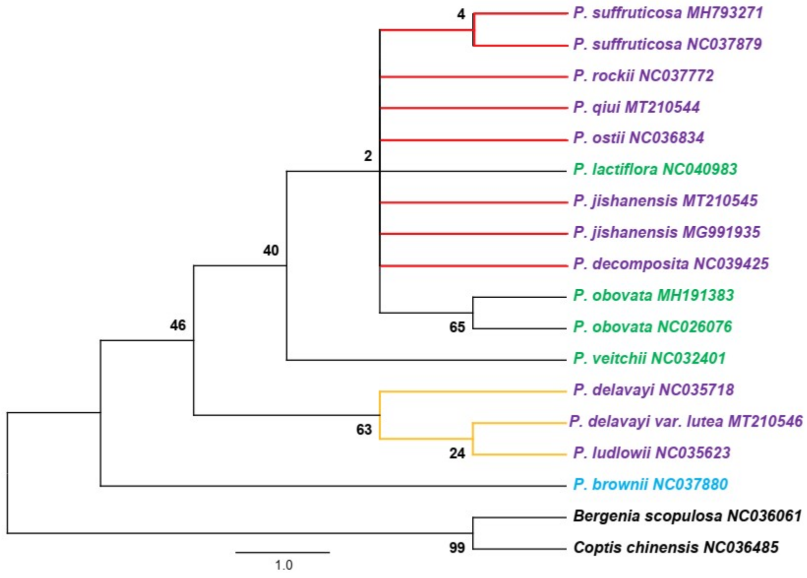

Supplementary Figure 2-1. Phylogenetic tree constructed using Maximum Likelihood (ML) method based on *psbZ* of chloroplast genomes of 16 *Paeonia* species. purple Latin names: species of sect. *Moutan*; blue Latin names: species of sect. *Onaepia*; green Latin names: species of sect. *Paeonia*; black Latin names: outgroups; red branches: species of subsect. *Vaginatae*; yellow branches: species of subsect. *Delavayanae*, the same below.

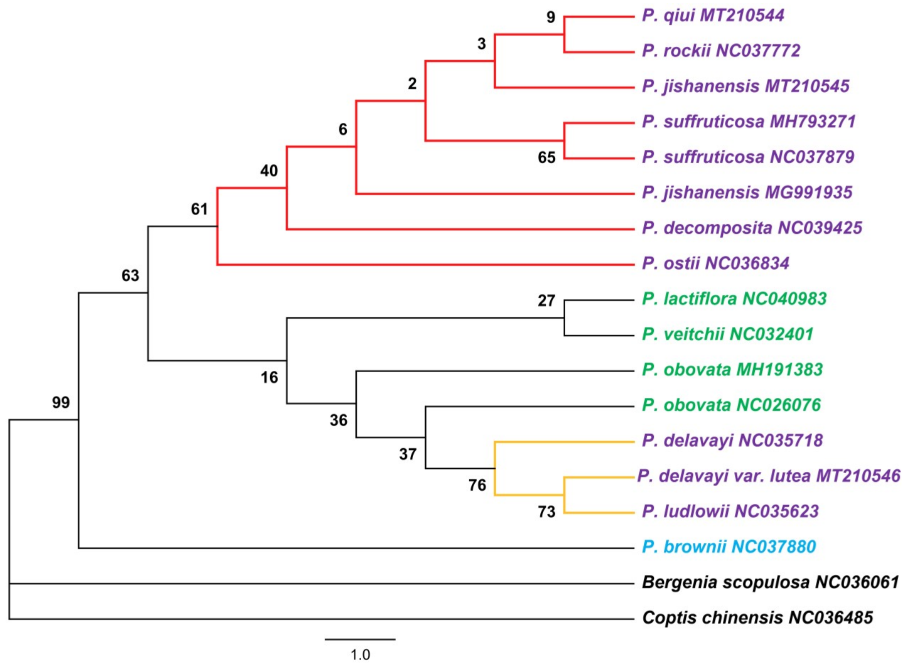

Supplementary Figure 2-2. Phylogenetic tree constructed using ML method based on *rps19* of chloroplast genomes of 16 *Paeonia* species.

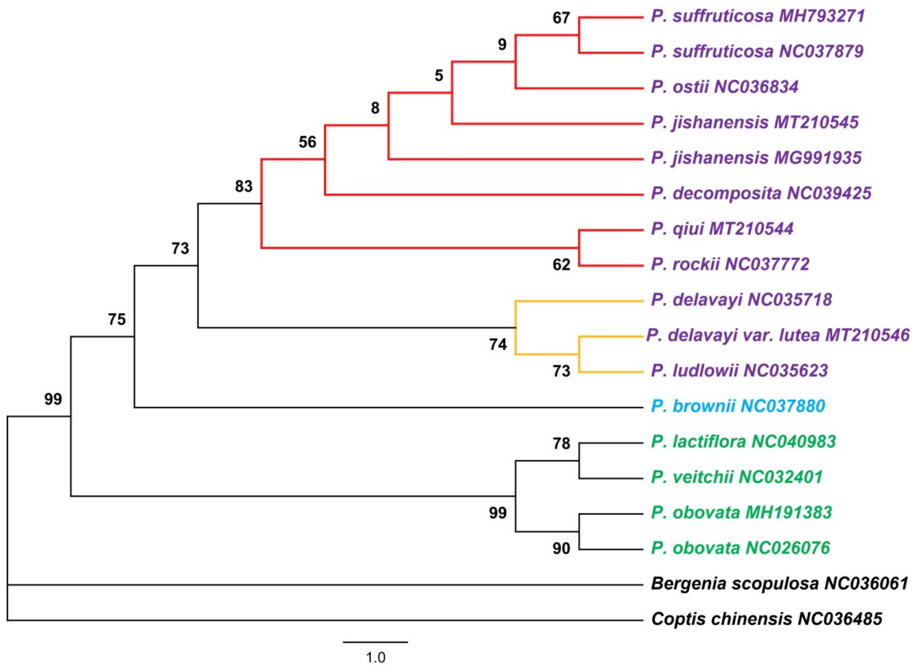

Supplementary Figure 2-3. Phylogenetic tree constructed using ML method based on *rps3* of chloroplast genomes of 16 *Paeonia* species.

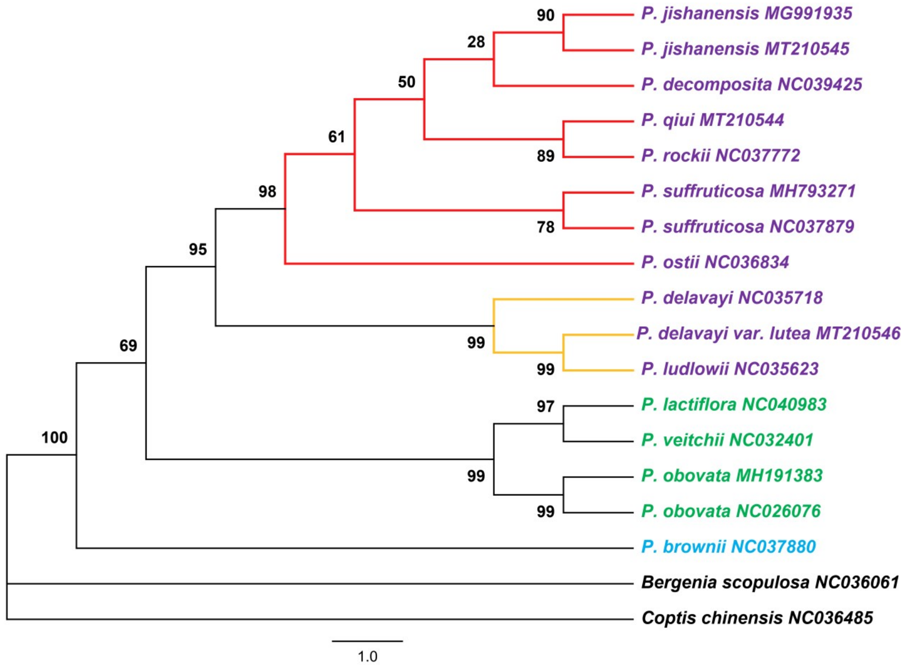

Supplementary Figure 2-4. Phylogenetic tree constructed using ML method based on *trnK* of chloroplast genomes of 16 *Paeonia* species.

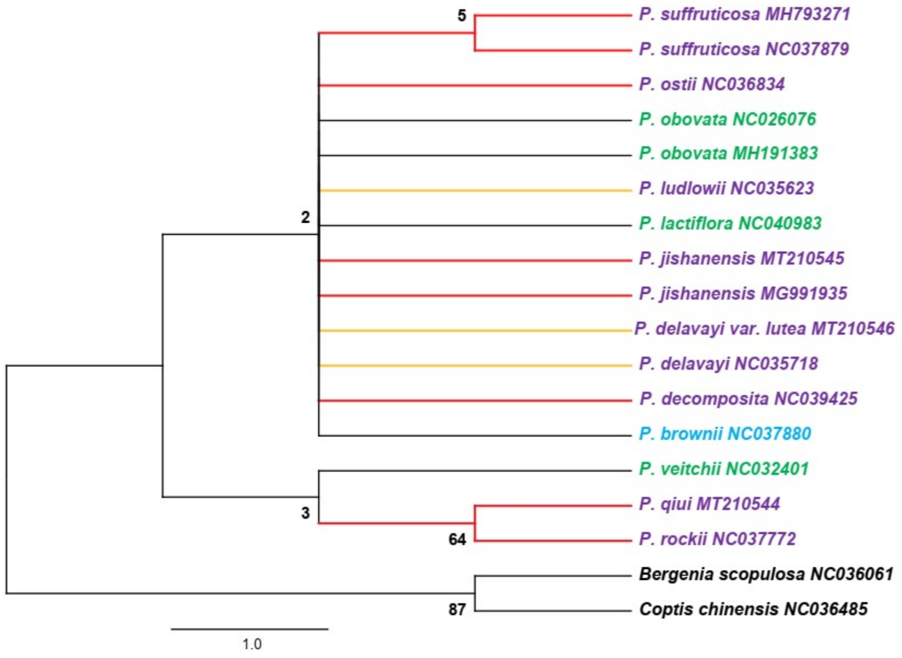

Supplementary Figure 2-5. Phylogenetic tree constructed using ML method based on *trnR* of chloroplast genomes of 16 *Paeonia* species.

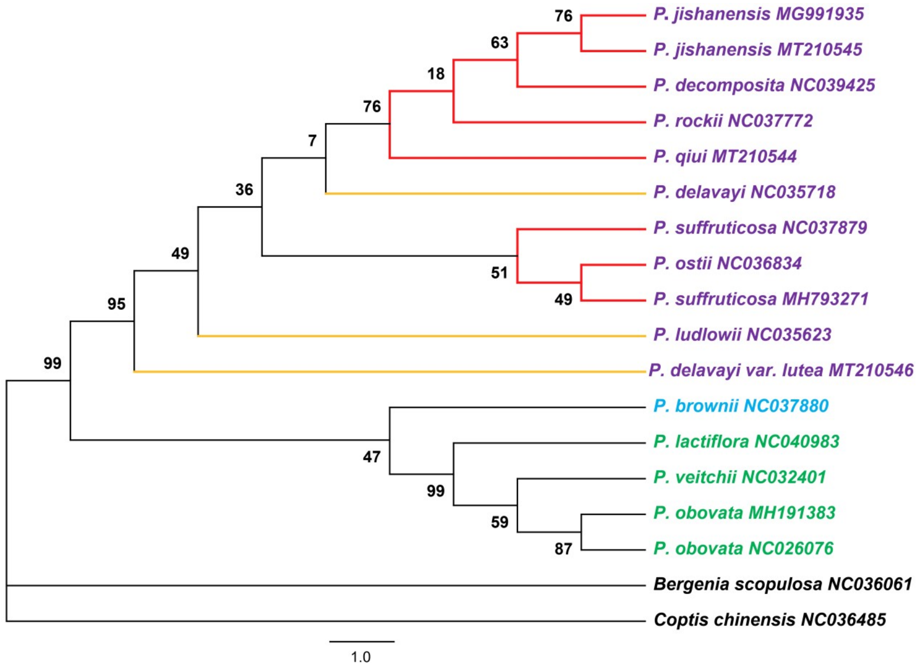

Supplementary Figure 2-6. Phylogenetic tree constructed using ML method based on *ycf3* of chloroplast genomes of 16 *Paeonia* species.

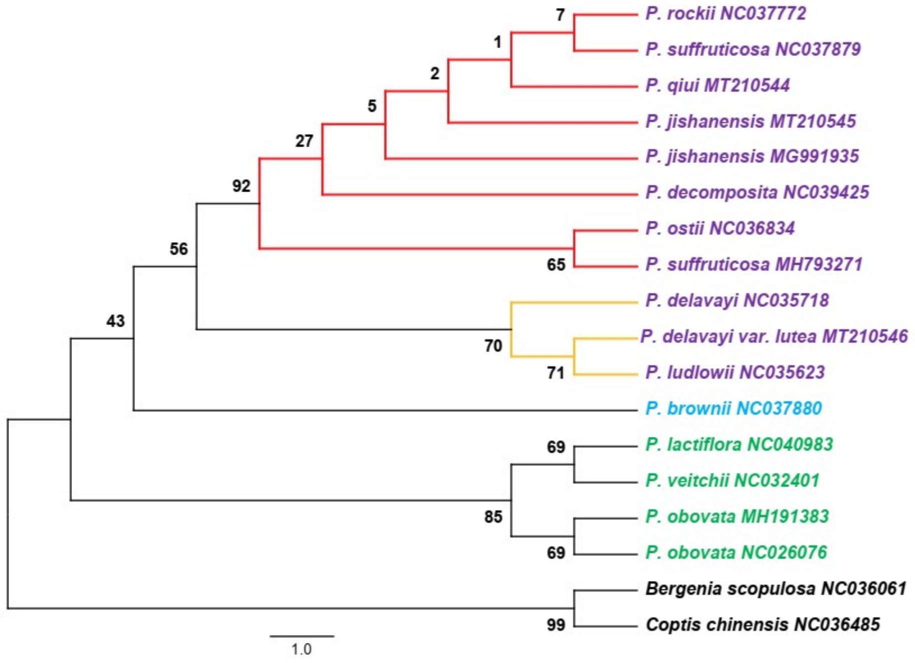

Supplementary Figure 2-7. Phylogenetic tree constructed using ML method based on *atpB-rbcL* of chloroplast genomes of 16 *Paeonia* species.

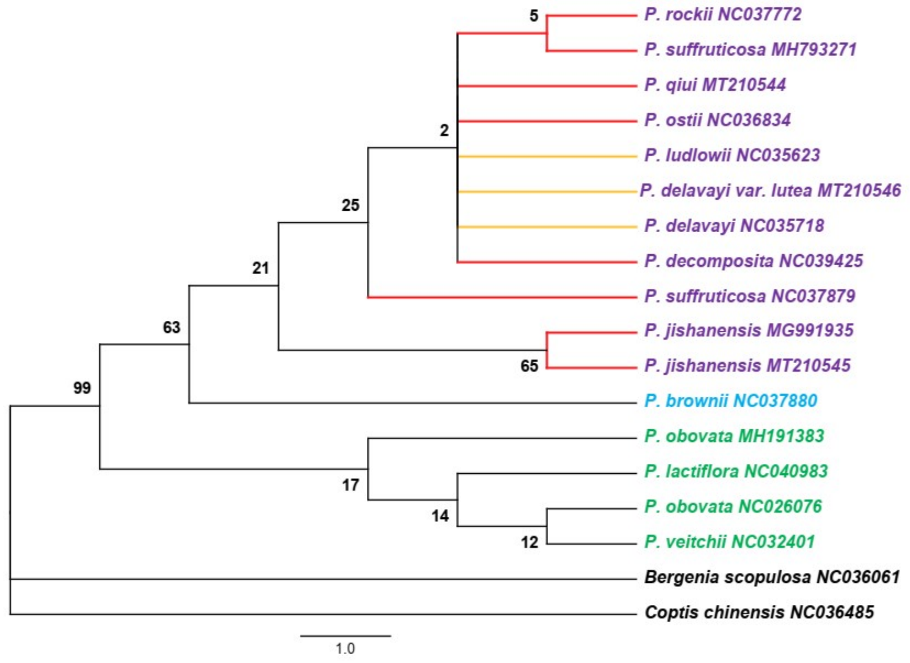

Supplementary Figure 2-8. Phylogenetic tree constructed using ML method based on *ndhB-trnL* of chloroplast genomes of 16 *Paeonia* species.

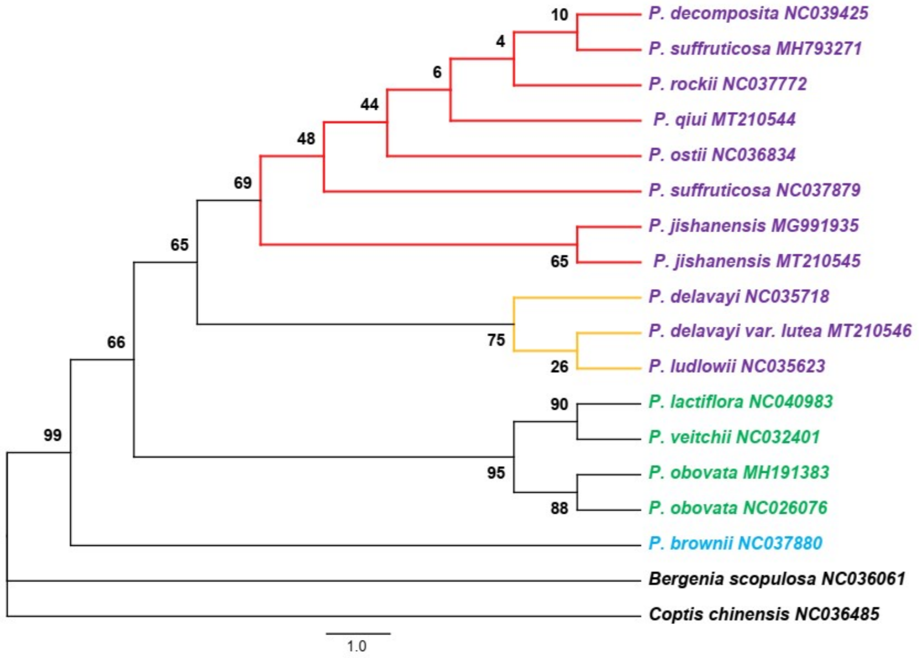

Supplementary Figure 2-9. Phylogenetic tree constructed using ML method based on *ndhC-trnV* of chloroplast genomes of 16 *Paeonia* species.

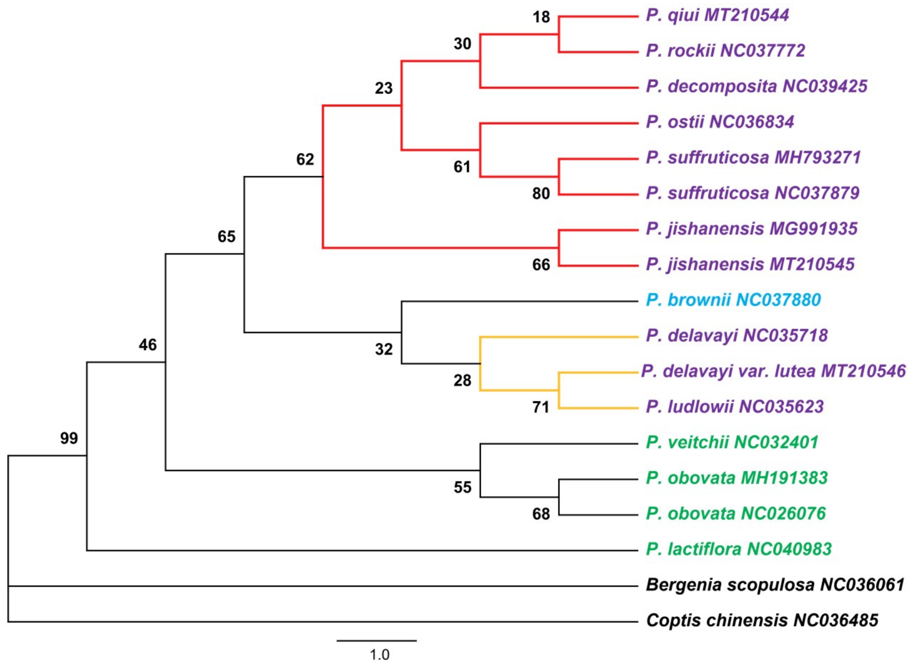

Supplementary Figure 2-10. Phylogenetic tree constructed using ML method based on *ndhG-ndhI* of chloroplast genomes of 16 *Paeonia* species.

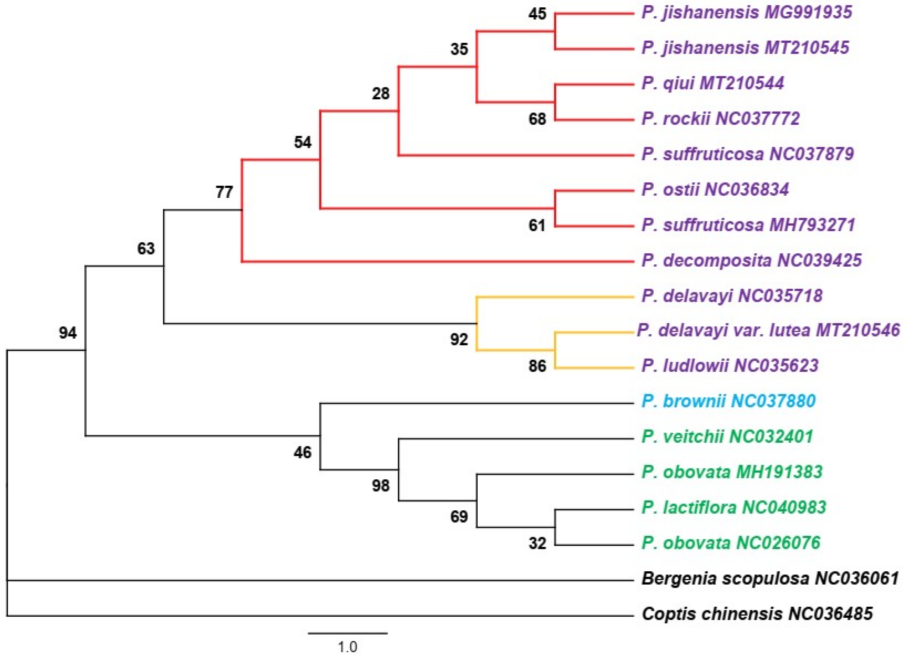

Supplementary Figure 2-11. Phylogenetic tree constructed using ML method based on *petA-psbJ* of chloroplast genomes of 16 *Paeonia* species.

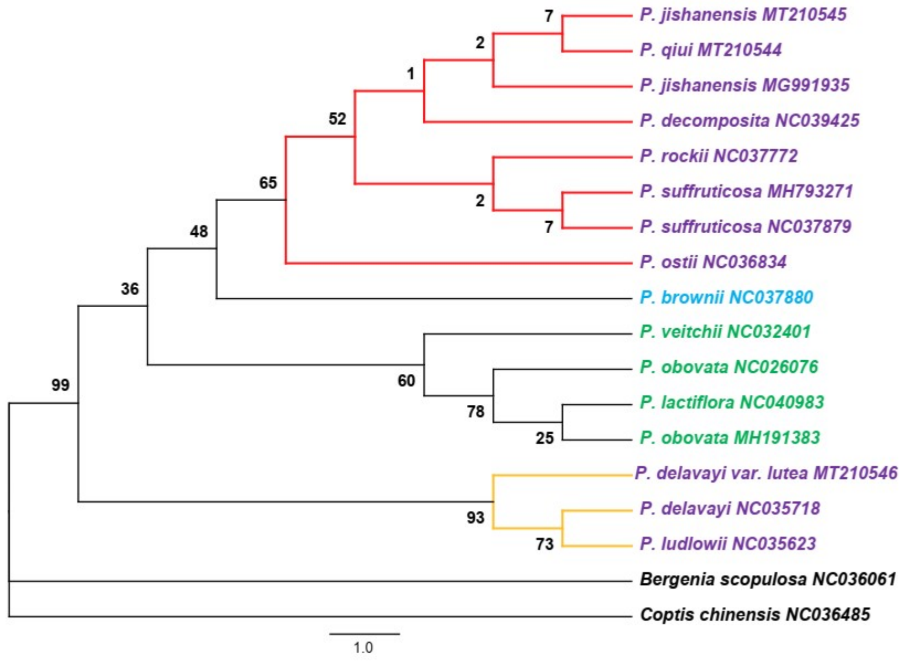

Supplementary Figure 2-12. Phylogenetic tree constructed using ML method based on *psbM-trnD* of chloroplast genomes of 16 *Paeonia* species.

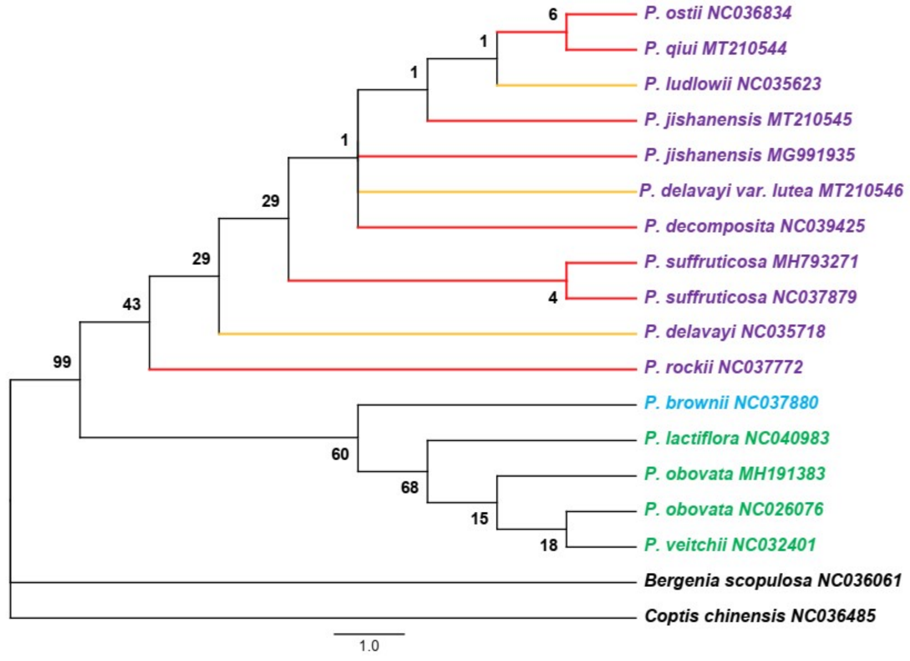

Supplementary Figure 2-13. Phylogenetic tree constructed using ML method based on *psbZ-trnG* of chloroplast genomes of 16 *Paeonia* species.

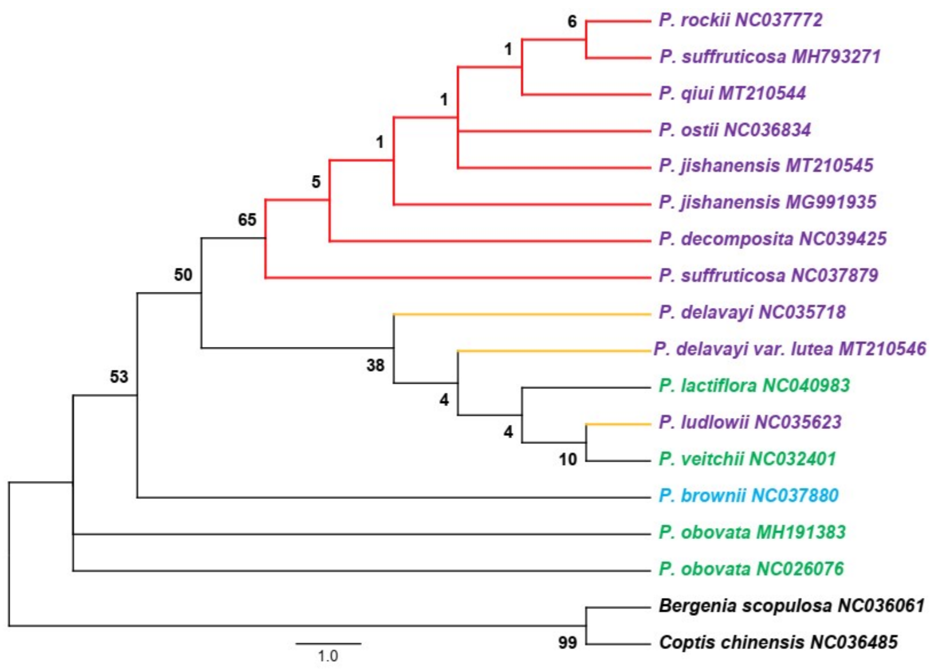

Supplementary Figure 2-14. Phylogenetic tree constructed using ML method based on *rpl16-rps3* of chloroplast genomes of 16 *Paeonia* species.

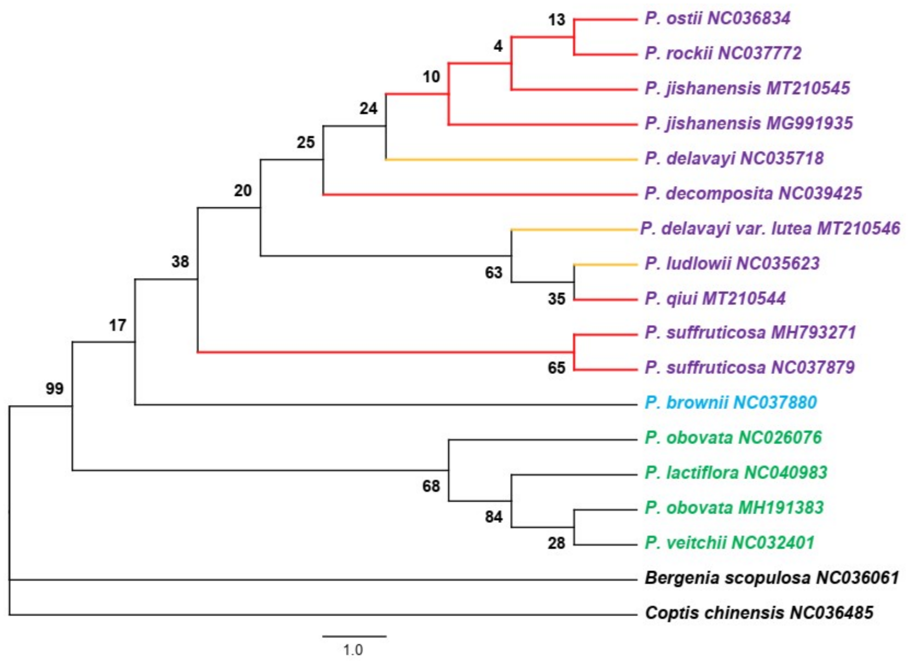

Supplementary Figure 2-15. Phylogenetic tree constructed using ML method based on *rpl20-rps12* of chloroplast genomes of 16 *Paeonia* species.

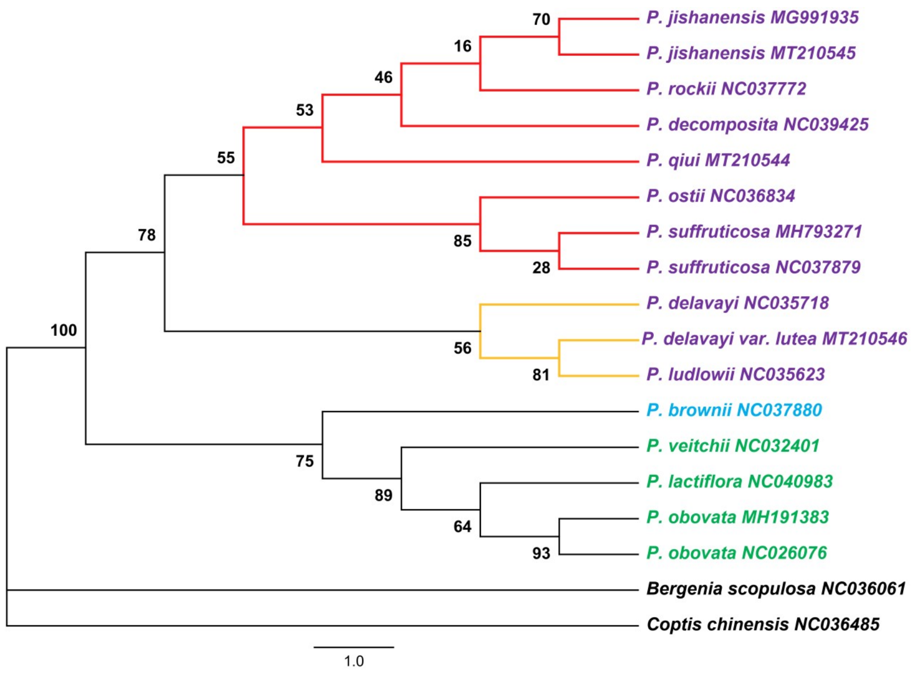

Supplementary Figure 2-16. Phylogenetic tree constructed using ML method based on *rpoB-trnC* of chloroplast genomes of 16 *Paeonia* species.

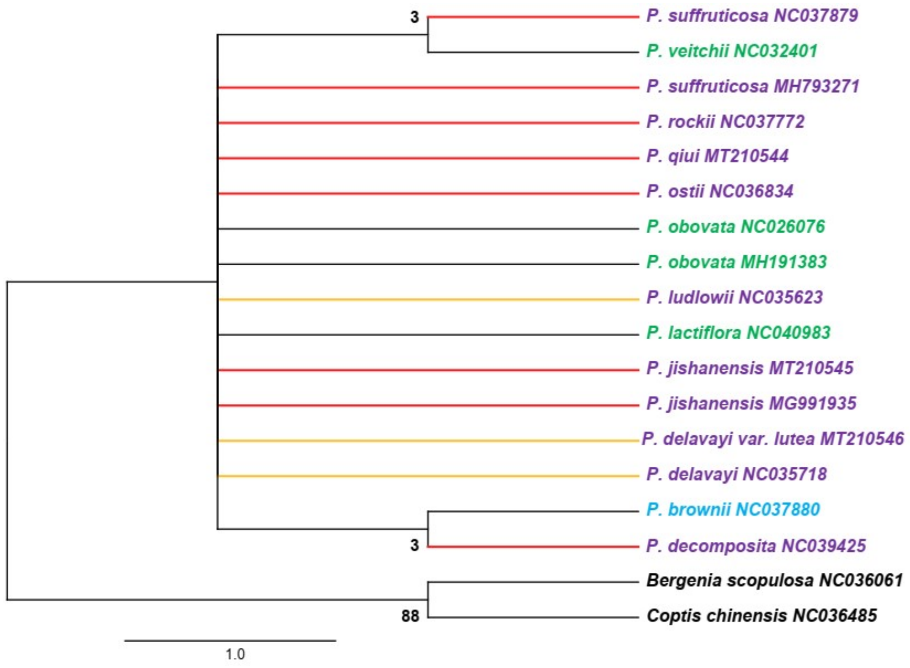

Supplementary Figure 2-17. Phylogenetic tree constructed using ML method based on *rpoC1-rpoB* of chloroplast genomes of 16 *Paeonia* species.

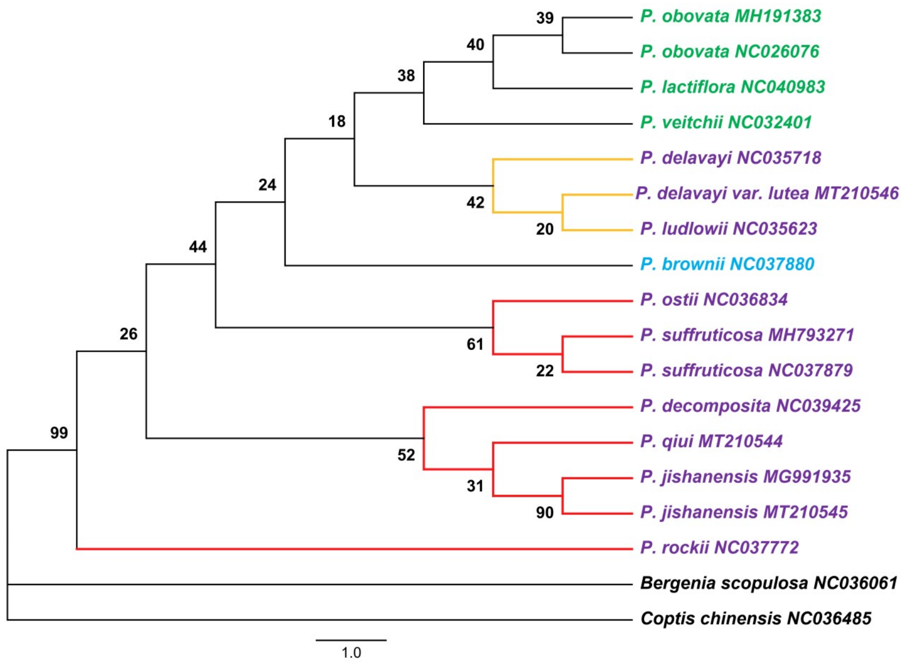

Supplementary Figure 2-18. Phylogenetic tree constructed using ML method based on *rps16-trnQ* of chloroplast genomes of 16 *Paeonia* species.

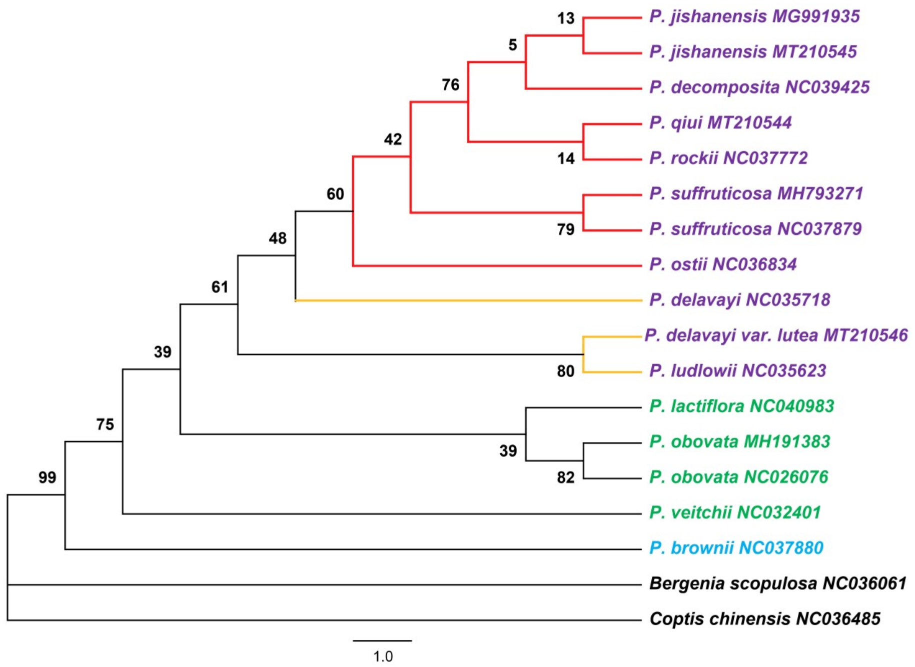

Supplementary Figure 2-19. Phylogenetic tree constructed using ML method based on *trnK-rps16* of chloroplast genomes of 16 *Paeonia* species.

1.2 Supplementary Tables

Supplementary Table 1. Information of the 16 *Paeonia* species.

No.	Species Name	Length /bp	Accession No.
1	*Paeonia brownii*	152,228	NC037880
2	*Paeonia decomposita*	152,601	NC039425
3	*Paeonia delavayi*	154,405	NC035718
4	*Paeonia delavayi* var. *lutea**	152,790	MT210546
5	*Paeonia jishanensis*	152,628	MG991935
6	*Paeonia jishanensis**	152,631	MT210545
7	*Paeonia lactiflora*	152,747	NC040983
8	*Paeonia ludlowii*	152,687	NC035623
9	*Paeonia obovata*	152,736	MH191383
10	*Paeonia obovata*	152,698	NC026076
11	*Paeonia ostii*	152,153	NC036834
12	*Paeonia qiui**	152,578	MT210544
13	*Paeonia rockii*	152,821	NC037772
14	*Paeonia suffruticosa*	152,811	MH793271
15	*Paeonia suffruticosa*	153,119	NC037879
16	*Paeonia veitchii*	152,682	NC032401

* Sequences from this study

Supplementary Table 2. The positions of the 19 highly variable regions.

No.	Regions	Start	End	Location
1	*trnK*	1722	4235	LSC
2	*trnK-rps16*	4236	4961	LSC
3	*rps16-trnQ*	6053	7090	LSC
4	*trnR*	9987	10058	LSC
5	*rpoC1-rpoB*	23902	23927	LSC
6	*rpoB-trnC*	27141	28095	LSC
7	*psbM-trnD*	29910	30989	LSC
8	*psbZ*	36548	36736	LSC
9	*psbZ-trnG*	36737	37058	LSC
10	*ycf3*	43067	45054	LSC
11	*ndhC-trnV*	51078	52080	LSC
12	*atpB-rbcL*	55038	55804	LSC
13	*petA-psbJ*	63319	64154	LSC
14	*rpl20-rps12*	68981	69745	LSC
15	*rpl16-rps3*	82548	82701	LSC
16	*rps3*	82702	83358	LSC
17	*rps19*	83966	84244	LSC
18	*ndhG-ndhI*	117665	118036	SSC
19	*ndhB-trnL*	142364	142915	IR

Supplementary Table 3-1. Codons in chloroplast genome of *P. qiui*.

Amino acid	Codon	No.	RSCU	tRNA	Amino acid	Codon	No.	RSCU	tRNA
Phe/F	UUU	923	1.27		Tyr/Y	UAU	767	1.62	
	UUC	532	0.73	*trnF-GAA*		UAC	181	0.38	*trnY-GUA*
Leu/L	UUA	837	1.86	*trnL-UAA*	TER	UAA	43	1.54	
	UUG	536	1.19	*trnL-CAA*		UAG	21	0.75	
	CUU	572	1.27		His/H	CAU	476	1.55	
	CUC	187	0.42			CAC	140	0.45	*trnH-GUG*
	CUA	377	0.84	*trnL-UAG*	Gln/Q	CAA	692	1.52	*trnQ-UUG*
	CUG	184	0.41			CAG	219	0.48	
Ile/I	AUU	1071	1.46		Asn/N	AAU	963	1.56	
	AUC	460	0.63	*trnI-GAU*		AAC	275	0.44	*trnN-GUU*
	AUA	675	0.92	*trnI-CAU*	Lys/K	AAA	1028	1.5	*trnK-UUU*
Met/M	AUG	612	1	*trn(f)M-CAU*		AAG	347	0.5	
Val/V	GUU	499	1.44		Asp/D	GAU	849	1.6	
	GUC	160	0.46	*trnV-GAC*		GAC	215	0.4	*trnD-GUC*
	GUA	522	1.51	*trnV-UAC*	Glu/E	GAA	980	1.48	*trnE-UUC*
	GUG	202	0.58			GAG	346	0.52	
Ser/S	UCU	538	1.64		Cys/C	UGU	217	1.49	
	UCC	335	1.02	*trnS-GGA*		UGC	74	0.51	*trnC-GCA*
	UCA	391	1.19	*trnS-UGA*	TER	UGA	20	0.71	
	UCG	193	0.59		Trp/W	UGG	455	1	*trnW-CCA*
Pro/P	CCU	408	1.47		Arg/R	CGU	324	1.26	*trnR-ACG*
	CCC	223	0.81			CGC	123	0.48	
	CCA	301	1.09	*trnP-UGG*		CGA	339	1.32	
	CCG	176	0.64			CGG	146	0.57	
Thr/T	ACU	506	1.54			AGA	381	1.16	*trnR-UCU*
	ACC	250	0.76	*trnT-GGU*		AGG	135	0.41	
	ACA	398	1.21	*trnT-UGU*	Ser/S	AGU	440	1.71	
	ACG	157	0.48			AGC	173	0.67	*trnS-GCU*
Ala/A	GCU	618	1.75		Gly/G	GGU	554	1.24	
	GCC	251	0.71			GGC	208	0.47	*trnG-GCC*
	GCA	368	1.04	*trnA-UGC*		GGA	689	1.54	*trnG-UCC*
	GCG	176	0.5			GGG	337	0.75	

Supplementary Table 3-2. Codons in chloroplast genome of *P. jishanensis*.

Amino acid	Codon	No.	RSCU	tRNA	Amino acid	Codon	No.	RSCU	tRNA
Phe/F	UUU	922	1.27		Tyr/Y	UAU	769	1.62	
	UUC	532	0.73	*trnF-GAA*		UAC	181	0.38	*trnY-GUA*
Leu/L	UUA	836	1.86	*trnL-UAA*	TER	UAA	43	1.54	
	UUG	537	1.19	*trnL-CAA*		UAG	21	0.75	
	CUU	572	1.27		His/H	CAU	475	1.54	
	CUC	187	0.42			CAC	140	0.46	*trnH-GUG*
	CUA	380	0.85	*trnL-UAG*	Gln/Q	CAA	692	1.52	*trnQ-UUG*
	CUG	185	0.41			CAG	219	0.48	
Ile/I	AUU	1071	1.46		Asn/N	AAU	964	1.56	
	AUC	461	0.63	*trnI-GAU*		AAC	274	0.44	*trnN-GUU*
	AUA	675	0.92	*trnI-CAU*	Lys/K	AAA	1026	1.49	*trnK-UUU*
Met/M	AUG	610	1	*trn(f)M-CAU*		AAG	347	0.51	
Val/V	GUU	495	1.43		Asp/D	GAU	850	1.6	
	GUC	161	0.47	*trnV-GAC*		GAC	214	0.4	*trnD-GUC*
	GUA	523	1.51	*trnV-UAC*	Glu/E	GAA	981	1.48	*trnE-UUC*
	GUG	203	0.59			GAG	346	0.52	
Ser/S	UCU	538	1.64		Cys/C	UGU	218	1.5	
	UCC	334	1.02	*trnS-GGA*		UGC	73	0.5	*trnC-GCA*
	UCA	391	1.19	*trnS-UGA*	TER	UGA	20	0.71	
	UCG	193	0.59		Trp/W	UGG	456	1	*trnW-CCA*
Pro/P	CCU	407	1.47		Arg/R	CGU	323	1.26	*trnR-ACG*
	CCC	225	0.81			CGC	123	0.48	
	CCA	301	1.09	*trnP-UGG*		CGA	338	1.31	
	CCG	173	0.63			CGG	146	0.57	
Thr/T	ACU	507	1.54			AGA	381	1.16	*trnR-UCU*
	ACC	250	0.76	*trnT-GGU*		AGG	136	0.41	
	ACA	398	1.21	*trnT-UGU*	Ser/S	AGU	440	1.71	
	ACG	158	0.48			AGC	173	0.67	*trnS-GCU*
Ala/A	GCU	617	1.75		Gly/G	GGU	557	1.25	
	GCC	251	0.71			GGC	207	0.46	*trnG-GCC*
	GCA	369	1.04	*trnA-UGC*		GGA	688	1.54	*trnG-UCC*
	GCG	176	0.5			GGG	336	0.75	

Supplementary Table 3-3. Codons in chloroplast genome of *P. delavayi* var. *lutea*.

Amino acid	Codon	No.	RSCU	tRNA	Amino acid	Codon	No.	RSCU	tRNA
Phe/F	UUU	895	1.27		Tyr/Y	UAU	740	1.61	
	UUC	518	0.73	*trnF-GAA*		UAC	182	0.39	*trnY-GUA*
Leu/L	UUA	816	1.86	*trnL-UAA*	TER	UAA	43	1.55	
	UUG	526	1.2	*trnL-CAA*		UAG	21	0.76	
	CUU	558	1.27		His/H	CAU	461	1.54	
	CUC	185	0.42			CAC	137	0.46	*trnH-GUG*
	CUA	368	0.84	*trnL-UAG*	Gln/Q	CAA	681	1.52	*trnQ-UUG*
	CUG	179	0.41			CAG	216	0.48	
Ile/I	AUU	1045	1.45		Asn/N	AAU	938	1.55	
	AUC	454	0.63	*trnI-GAU*		AAC	271	0.45	*trnN-GUU*
	AUA	659	0.92	*trnI-CAU*	Lys/K	AAA	1002	1.49	*trnK-UUU*
Met/M	AUG	608	1	*trn(f)M-CAU*		AAG	339	0.51	
Val/V	GUU	488	1.43		Asp/D	GAU	831	1.59	
	GUC	165	0.48	*trnV-GAC*		GAC	213	0.41	*trnD-GUC*
	GUA	515	1.51	*trnV-UAC*	Glu/E	GAA	964	1.48	*trnE-UUC*
	GUG	193	0.57			GAG	340	0.52	
Ser/S	UCU	520	1.63		Cys/C	UGU	213	1.47	
	UCC	330	1.03	*trnS-GGA*		UGC	76	0.53	*trnC-GCA*
	UCA	372	1.16	*trnS-UGA*	TER	UGA	19	0.69	
	UCG	191	0.6		Trp/W	UGG	446	1	*trnW-CCA*
Pro/P	CCU	398	1.45		Arg/R	CGU	318	1.27	*trnR-ACG*
	CCC	225	0.82			CGC	122	0.49	
	CCA	296	1.08	*trnP-UGG*		CGA	324	1.29	
	CCG	177	0.65			CGG	145	0.58	
Thr/T	ACU	499	1.53			AGA	372	1.16	*trnR-UCU*
	ACC	249	0.76	*trnT-GGU*		AGG	135	0.42	
	ACA	396	1.22	*trnT-UGU*	Ser/S	AGU	433	1.72	
	ACG	158	0.49			AGC	166	0.66	*trnS-GCU*
Ala/A	GCU	601	1.72		Gly/G	GGU	549	1.24	
	GCC	251	0.72			GGC	209	0.47	*trnG-GCC*
	GCA	368	1.05	*trnA-UGC*		GGA	679	1.53	*trnG-UCC*
	GCG	176	0.5			GGG	333	0.75	
